# Early Functional Outcomes and Recovery of Hand Strength After Carpal Tunnel Release

**DOI:** 10.7759/cureus.107461

**Published:** 2026-04-21

**Authors:** Evangelos Tzanis, Sophia Syngouna, Anastassios Philippou, Evangelos Sakellariou, John Vlamis

**Affiliations:** 1 3rd Orthopaedic Department, KAT General Hospital of Attica, School of Medicine, National and Kapodistrian University of Athens, Athens, GRC; 2 Hand-Upper Limb and Microsurgery Department, KAT General Hospital of Attica, Kifisia, GRC; 3 Physiology Department, School of Medicine, National and Kapodistrian University of Athens, Athens, GRC

**Keywords:** carpal tunnel release, carpal tunnel syndrome, functional recovery, grip strength, pinch strength, postoperative outcomes

## Abstract

Background: Although carpal tunnel release reliably improves symptoms in patients with carpal tunnel syndrome (CTS), the short-term pattern of postoperative recovery in hand strength remains incompletely characterized. Serial measurement of grip and pinch strength, together with patient-reported outcome assessment, may provide a more clinically meaningful description of early recovery after surgery.

Methods: This prospective observational cohort study included 21 adults with clinically and electrophysiologically confirmed CTS who underwent standardized mini-open carpal tunnel release. All participants followed the same structured 12-week home-based postoperative strengthening protocol beginning in the third postoperative week, following the initial wound-healing period. Grip strength (Jamar hydraulic hand dynamometer) and pinch strength were measured preoperatively and at three, nine, and 15 weeks postoperatively. Symptom severity and functional status were assessed using the Boston Carpal Tunnel Questionnaire (BCTQ) preoperatively and at 15 weeks postoperatively. Longitudinal changes in grip and key pinch strength were analyzed using repeated-measures analysis of variance, while changes in BCTQ symptom severity and functional status scores from baseline to 15 weeks were analyzed using paired-samples t-tests.

Results: Grip and key pinch strength demonstrated an early reduction during the postoperative period, followed by progressive improvement at subsequent follow-up assessments. Grip strength declined at three weeks, returned toward preoperative values by nine weeks, and exceeded preoperative values by 15 weeks postoperatively (27.52 ± 9.22 kg preoperatively vs. 32.33 ± 7.77 kg at 15 weeks; p < 0.001), while key pinch strength showed a similar recovery pattern over time (7.33 ± 1.77 kg preoperatively vs. 8.10 ± 1.86 kg at 15 weeks; p < 0.001). Patient-reported outcomes demonstrated marked improvement, with significant reductions in BCTQ symptom severity scores (3.22 ± 0.55 to 1.00 ± 0.00; p < 0.001) and functional status scores (2.79 ± 0.62 to 1.02 ± 0.08; p < 0.001) at follow-up.

Conclusions: Early postoperative recovery following carpal tunnel release is characterized by a transient decline in grip and key pinch strength at three weeks, return toward baseline by nine weeks, and improvement beyond baseline by 15 weeks. Objective strength measures and patient-reported outcomes provide complementary information regarding recovery dynamics and may assist clinicians in monitoring early postoperative recovery after carpal tunnel release.

## Introduction

Carpal tunnel syndrome (CTS) is the most frequently encountered entrapment neuropathy of the upper extremity and can lead to clinically relevant limitations in hand use [1]. It typically presents with pain, paresthesia, and sensory disturbances, while advanced cases may also demonstrate reduced muscle strength and diminished hand function [1-3]. Beyond sensory symptoms, CTS may cause measurable functional deficits that interfere with daily and occupational activities, making functional limitation an important reason for treatment seeking and surgical decision-making [1-3].

For patients with persistent symptoms or more advanced disease, surgical decompression is widely used and is generally associated with substantial symptom improvement [4-6]. In addition, clinical and neurophysiological recovery after carpal tunnel release has been documented in prior studies [7]. Nevertheless, despite adequate decompression of the median nerve, postoperative recovery is not always linear in terms of hand function. Patients may experience transient reductions in hand strength and functional capacity during the early postoperative period [7-9]. These findings suggest that, although carpal tunnel release is effective for neural decompression and symptom relief, recovery of hand function during the early postoperative period may remain variable.

Despite adequate median nerve decompression, restoration of hand strength does not typically occur immediately after surgery and may follow a staged recovery pattern [7-10]. Early postoperative assessments have shown that grip and pinch strength may transiently decline before demonstrating gradual improvement over time [7,9]. This pattern is clinically relevant, as temporary reductions in hand strength can interfere with functional performance and may delay return to daily and occupational activities [7,8]. Consequently, postoperative muscle strength represents a critical component of functional recovery assessment following carpal tunnel release.

Assessment of postoperative recovery should incorporate both objective performance measures and patient-reported outcomes. Grip and pinch testing provide practical markers of hand performance, whereas disease-specific questionnaires help quantify symptom burden and perceived functional limitation [11-17]. The Boston Carpal Tunnel Questionnaire (BCTQ) is among the most commonly used disease-specific tools for quantifying symptom severity and functional limitation in CTS [15,16], while the Greek version has also been shown to be a valid and reliable instrument for use in Greek-speaking patients with CTS [17]. The integration of objective strength assessments with patient-reported outcome tools is therefore essential for a comprehensive evaluation of postoperative recovery [11,15,16].

However, published reports differ considerably in surgical technique (open, mini-open, or extended open release), follow-up timing, postoperative splinting or rehabilitation protocols, and the selection of strength and patient-reported outcome measures, making early postoperative functional recovery difficult to interpret across studies [7-10,18-23]. A recent systematic review likewise found no consistent benefit of routine postoperative splinting after carpal tunnel release, suggesting that immobilization should not be used indiscriminately [24]. In addition, hand rehabilitation programs have been reported to improve functional outcomes after carpal tunnel surgery, although the magnitude of benefit appears to vary across studies [25]. This variability limits the ability to draw consistent clinical conclusions regarding early postoperative recovery. In addition, studies that combine serial objective strength measurements with functional questionnaire outcomes at predefined postoperative intervals remain relatively limited [9,11]. Therefore, the aim of the present study was to evaluate postoperative functional recovery following carpal tunnel release, with particular emphasis on serial changes in grip strength and pinch strength and patient-reported functional outcomes in patients managed with a standardized postoperative rehabilitation protocol.

## Materials and methods

Study design

This prospective observational cohort study was designed to evaluate functional recovery following the surgical treatment of CTS. The study was conducted at KAT General Hospital of Attica, Athens, Greece, from April 27, 2022, to July 9, 2025. All assessments were performed at predefined time points before and after surgery to document serial changes in hand strength and patient-reported functional outcomes. All participants were managed postoperatively using the same structured strengthening protocol after the initial postoperative recovery phase.

Ethical approval

This study was conducted as part of an approved doctoral dissertation research protocol at the National and Kapodistrian University of Athens School of Medicine and KAT General Hospital of Attica, Athens, Greece. Institutional approval was obtained prior to participant enrollment from the Scientific Council and the Unified Administrative Board of KAT General Hospital of Attica (approval numbers: 681/06-07-2021 and 24/03-08-2021; final institutional approval date: August 3, 2021). All procedures were performed in accordance with the Declaration of Helsinki, and written informed consent was obtained from all participants.

Participants

The study included adult patients (>18 years) with idiopathic CTS, clinically diagnosed on the basis of medical history, physical examination, and provocative testing (including Phalen and Tinel tests), with electrodiagnostic confirmation by upper-limb electromyography in accordance with established guidelines. Electrodiagnostic evaluation was used to confirm the diagnosis and assess clinical severity; however, formal electrophysiological severity classification was not incorporated into the present statistical analysis. All patients had symptoms for at least six months and had failed conservative treatment prior to surgery. All participants completed a detailed medical and physical activity history prior to enrollment. Patients with previous carpal tunnel surgery in the affected hand, a history of wrist or hand trauma or carpal bone fracture, concurrent neurological disorders affecting upper-limb function (including polyneuropathy, proximal median or ulnar neuropathy, brachial plexopathy, multiple mononeuropathy, or cervical spondylosis), pregnancy or postpartum status, thyroid disease, rheumatoid arthritis, and diabetes mellitus were excluded.

Preoperative assessment

Before surgery, all patients underwent a baseline evaluation, including completion of the BCTQ and objective assessment of hand strength. Grip strength was assessed with a Jamar hydraulic hand dynamometer using the second handle position for all participants to ensure consistency across repeated assessments [14]. Key pinch strength was assessed using a pinch gauge, following established methods for grip and pinch strength evaluation [12]. All strength tests were performed under standardized positioning (seated, shoulder adducted, elbow flexed to 90°, and forearm in neutral position). Three trials were obtained for each measure, separated by 60 seconds of rest, and the mean value was used for analysis.

Outcome measures

Functional recovery was assessed using standardized objective measurements of hand strength and a validated patient-reported outcome measure. Grip strength and key pinch strength were selected as the primary objective outcomes [11-14], while symptom severity and functional status were assessed using the BCTQ, a validated disease-specific questionnaire originally developed for CTS [15], with additional clinical application reported in subsequent studies [16], including its validated Greek version [17]. Higher BCTQ scores indicate greater symptom severity and functional limitation.

Surgical procedure

All surgical procedures were performed by the same orthopedic surgeon using a standardized mini-open carpal tunnel release technique to minimize procedural variability. Local anesthesia was achieved with the subcutaneous infiltration of 5-7 mL of 1.0% lidocaine without epinephrine at the level of the distal wrist crease. A small palmar incision was made, and careful dissection was carried out to minimize trauma to surrounding soft tissues and reduce the risk of median nerve injury. Complete release of the transverse carpal ligament was achieved in all cases [18,19].

Postoperative management

Postoperative management followed a standardized protocol after carpal tunnel release, without routine immobilization beyond the immediate postoperative period [20,22,23]. Early mobilization of the wrist and hand was encouraged to facilitate functional use and reduce postoperative stiffness [20,22,23]. Patients received standardized instructions regarding gradual return to daily activities during the early postoperative phase.

Exercise protocol

The structured home-based strengthening protocol was initiated at the third postoperative week, following the initial postoperative recovery phase, and was continued for 12 weeks. The protocol included low-load resistance exercises targeting the wrist flexor and extensor muscles using 1-kg dumbbells, selected to provide a simple and standardized home-based loading approach during the early postoperative recovery period. Each dumbbell exercise was performed for three sets of 12 repetitions, once daily, four times per week, with a one-minute rest interval between sets. Additional exercises using a foam ball were included to support grip function and were performed in three sets of 10 repetitions. Patients received standardized verbal and written instructions regarding the home-based exercise program. Formal adherence to the home-based protocol was not quantitatively recorded, and this should be considered a limitation regarding reproducibility. Pain intensity during exercise was monitored using the numeric rating scale as a clinical safety and tolerance measure during follow-up; however, these data were not included in the present quantitative analysis.

Follow-up assessments

At three weeks postoperatively, patients underwent reassessment of grip and key pinch strength using identical testing conditions. At nine weeks postoperatively, grip and key pinch strength measurements were repeated. Patient-reported outcomes using the BCTQ were assessed only at baseline and at 15 weeks postoperatively; no intermediate BCTQ assessments were performed during follow-up. At 15 weeks postoperatively, following the completion of the strengthening program, all strength measurements were repeated, and participants again completed the BCTQ to assess functional outcomes. Additional follow-up data beyond 15 weeks were available in a subset of patients; however, these were not included in the quantitative analysis because of variability in follow-up duration.

Statistical analysis

Continuous variables were presented as mean ± standard deviation (SD) or median (interquartile range (IQR)), while categorical variables were expressed as frequencies (n) and percentages (%). The normality of data distribution was assessed using the Shapiro-Wilk test.

To evaluate the stability of the findings, a sensitivity analysis was performed using both parametric and non-parametric statistical methods. Longitudinal comparisons of grip strength and key pinch strength across the predefined assessment time points were performed using one-way repeated-measures analysis of variance (repeated-measures ANOVA), with the Bonferroni correction applied for pairwise comparisons. Longitudinal comparisons of BCTQ symptom severity and functional status scores between the two assessment time points were performed using the paired-samples t-test. For non-parametric analyses, the Friedman test and Wilcoxon signed-rank test were used.

Because of the exploratory prospective design and the available clinical cohort, a formal a priori sample size calculation was not performed.

All statistical analyses were conducted using IBM SPSS Statistics for Windows, V. 21.0 (IBM Corp., Armonk, NY, USA). All tests were two-sided, and a p-value of <0.05 was considered statistically significant.

## Results

Baseline characteristics of the study participants

The study sample consisted of 21 participants with a mean age of 52.52 ± 10.63 years. Regarding sex distribution, 10 participants (47.6%) were male, and 11 (52.4%) were female. The mean body weight was 79.86 ± 14.91 kg, the mean height was 168.19 ± 8.04 cm, and the mean body mass index was 28.16 ± 4.16 kg/m². Most participants were right-hand dominant (20 participants, 95.2%), whereas one participant (4.8%) was left-hand dominant. Surgery was performed on the right hand in 16 participants (76.2%) and on the left hand in five participants (23.8%) (Table 1). 

**Table 1:** Baseline characteristics of the study participants Continuous variables are presented as mean ± SD and categorical variables as n (%).

Variable	Value
Age (years), mean ± SD	52.52 ± 10.63
Sex, male/female, n (%)	10 (47.6%)/11 (52.4%)
Weight (kg), mean ± SD	79.86 ± 14.91
Height (cm), mean ± SD	168.19 ± 8.04
BMI (kg/m²), mean ± SD	28.16 ± 4.16
Dominant hand, right/left, n (%)	20 (95.2%)/1 (4.8%)
Operated hand, right/left, n (%)	16 (76.2%)/5 (23.8%)

Grip and pinch strength

Table 2 summarizes the parametric longitudinal analysis of grip strength and pinch strength across the predefined assessment time points. Because Mauchly's test of sphericity was statistically significant and the epsilon value was <0.75, the Greenhouse-Geisser correction was applied. The values shown in the columns labeled "3 weeks", "9 weeks", and "15 weeks" represent Bonferroni-adjusted pairwise comparison p-values between each row time point and the corresponding postoperative assessment time point.

**Table 2:** Longitudinal comparison of grip and pinch strength across the predefined assessment time points (parametric analysis) Data are presented as mean ± SD. Repeated-measures ANOVA with Greenhouse-Geisser correction was applied because the assumption of sphericity was violated. Values in the columns labeled "3 weeks", "9 weeks", and "15 weeks" represent Bonferroni-adjusted pairwise comparison p-values between the row time point and the corresponding postoperative assessment time point. A p-value of <0.05 was considered statistically significant. ANOVA: analysis of variance

Variable	Time point	Mean ± SD	3 weeks	9 weeks	15 weeks
Grip strength	Preoperative	27.52 ± 9.22	<0.001	1.000	<0.001
	3 weeks	22.29 ± 8.65	-	<0.001	<0.001
	9 weeks	28.00 ± 8.23	-	-	<0.001
	15 weeks	32.33 ± 7.77	-	-	-
	Repeated-measures ANOVA	F(1.91, 38.16) = 77.71; p < 0.001	Ν/Α	Ν/Α	Ν/Α
Pinch strength	Preoperative	7.33 ± 1.77	<0.001	1.000	<0.001
	3 weeks	6.45 ± 1.55	-	<0.001	<0.001
	9 weeks	7.40 ± 1.88	-	-	<0.001
	15 weeks	8.10 ± 1.86	-	-	-
	Repeated-measures ANOVA	F(2.41, 48.17) = 54.54; p < 0.001	Ν/Α	Ν/Α	Ν/Α

**Figure 1:**
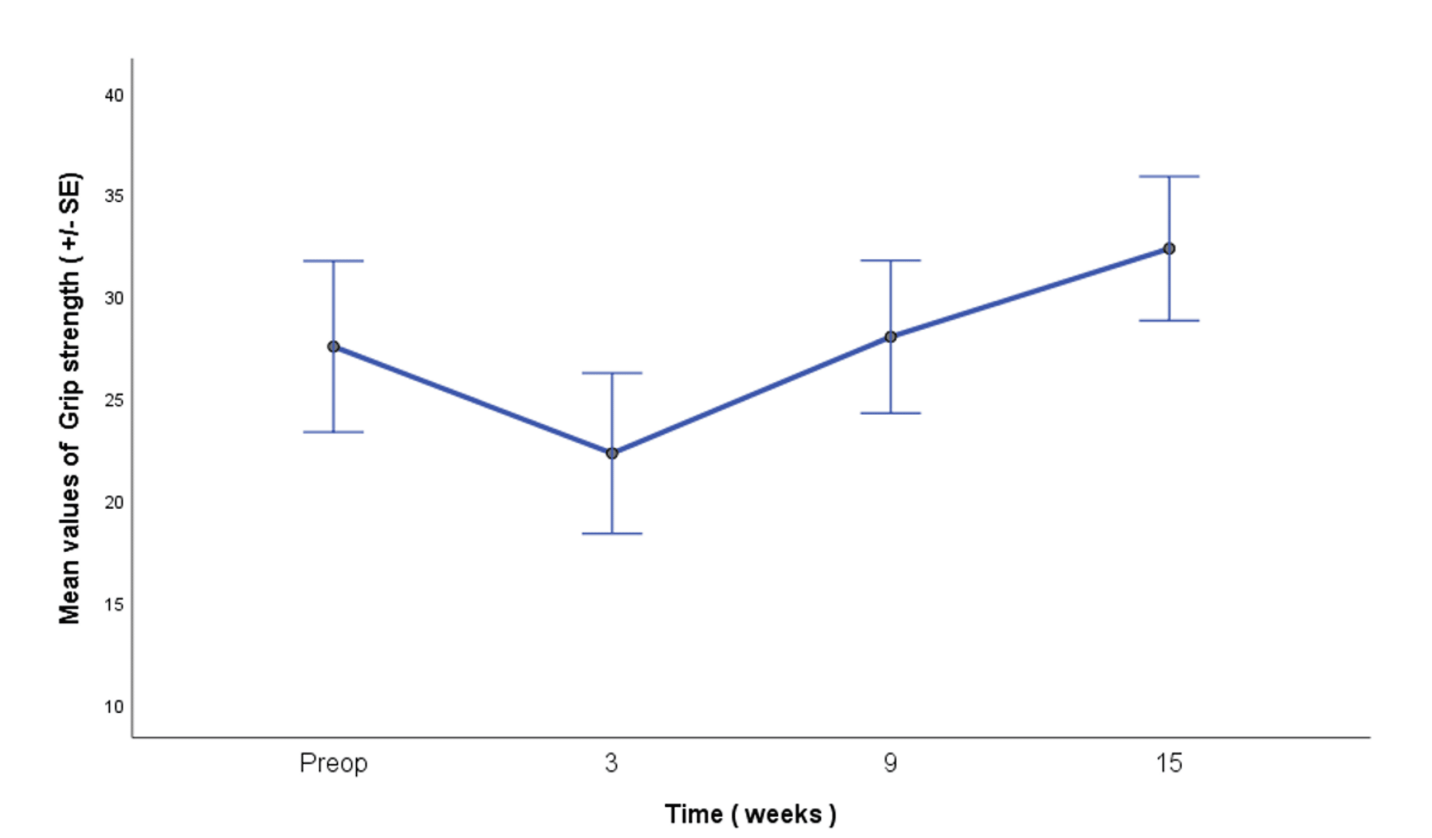
Grip strength across the predefined assessment time points

A statistically significant effect of time was observed for grip strength (F(1.91, 38.16) = 77.71; p < 0.001; partial η² = 0.795), indicating a large effect size. Pairwise comparisons demonstrated significant differences between all assessment time points, except between the preoperative and nine-week assessments (p = 1.000). This finding indicates that grip strength had returned to approximately baseline levels by nine weeks following the early postoperative decline observed at three weeks and subsequently exceeded baseline values at 15 weeks (Figure 1).

**Figure 2:**
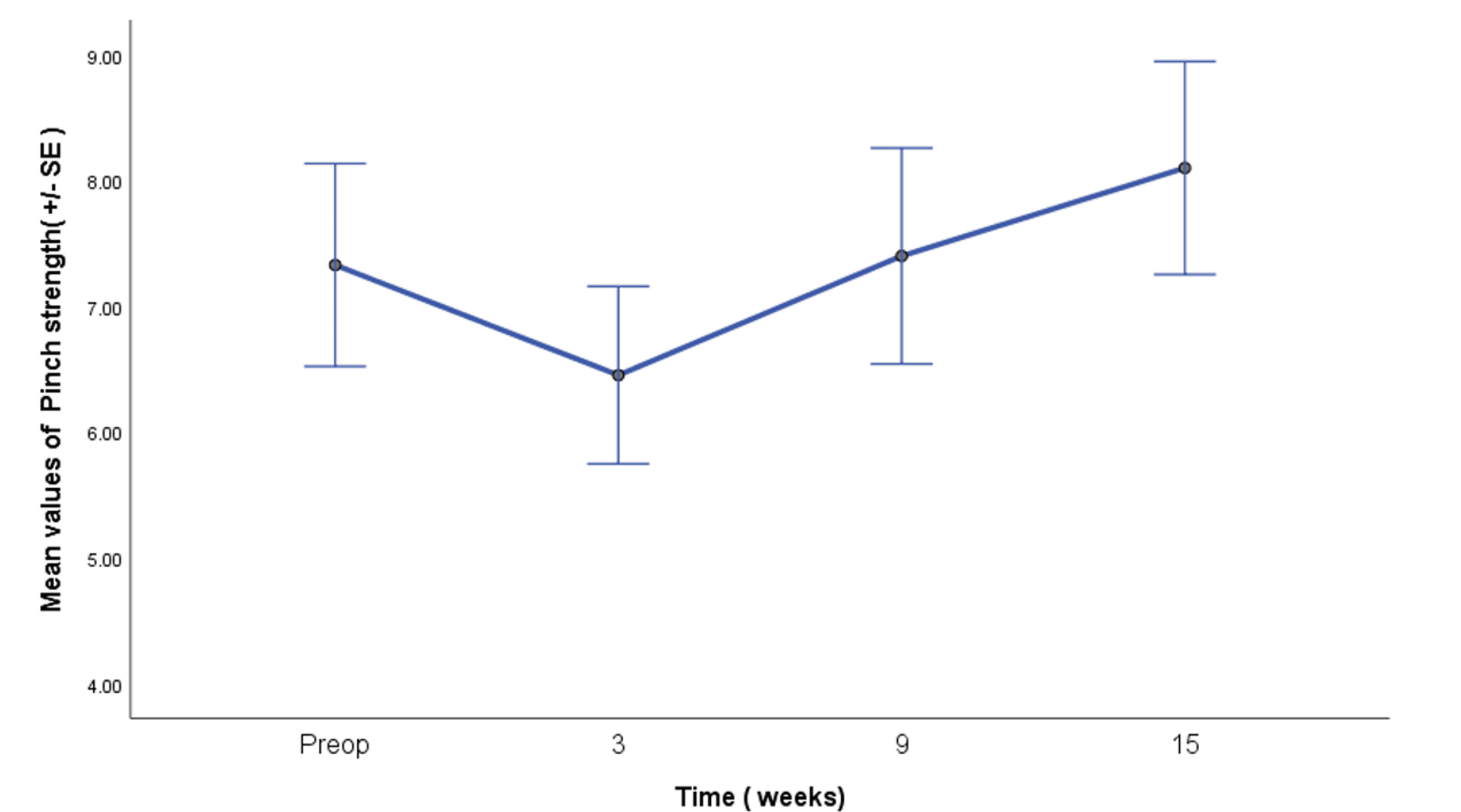
Pinch strength across the predefined assessment time points

A statistically significant effect of time was also observed for pinch strength (F(2.41, 48.17) = 54.54; p < 0.001; partial η² = 0.732), also indicating a large effect size. Bonferroni-adjusted pairwise comparisons similarly demonstrated significant differences between all time points, except between the preoperative and nine-week assessments (adjusted p = 1.000). This pattern likewise indicates that key pinch strength had recovered to approximately baseline levels by nine weeks after the initial postoperative decline and improved further by 15 weeks (Figure 2).

BCTQ outcomes

Table 3 presents the comparison of BCTQ symptom severity and functional status scores between baseline and 15 weeks postoperatively.

**Table 3:** Comparison of BCTQ symptom severity and functional status scores between baseline and 15 weeks postoperatively Parametric results are presented as mean ± SD, and differences are expressed as mean difference with 95% CI. Non-parametric results are presented as median (IQR), and differences are expressed as median difference (IQR). A p-value of <0.05 was considered statistically significant. BCTQ: Boston Carpal Tunnel Questionnaire; IQR: interquartile range; CI: confidence interval

Parametric	Mean ± SD (baseline)	Mean ± SD (15 weeks)	Mean difference (95% CI)	P-value
BCTQ-symptoms	3.22 ± 0.55	1.00 ± 0.00	-2.22 (-2.46 to -1.97)	<0.001
BCTQ-functional	2.79 ± 0.62	1.02 ± 0.08	-1.77 (-2.05 to -1.50)	<0.001
Non-parametric	Median (IQR) (baseline)	Median (IQR) (15 weeks)	Median difference (IQR)	P-value
BCTQ-symptoms	3.36 (0.82)	1.00 (0.0)	-2.36 (0.82)	<0.001
BCTQ-functional	2.86 (1.06)	1.00 (0.0)	-1.87 (1.06)	<0.001

In the primary parametric analysis, both BCTQ subscales showed statistically significant improvement at 15 weeks. BCTQ symptom severity decreased from 3.22 ± 0.55 at baseline to 1.00 ± 0.00 at 15 weeks, corresponding to a mean difference of −2.22 (95% CI: −2.46 to −1.97; p < 0.001). BCTQ functional status decreased from 2.79 ± 0.62 to 1.02 ± 0.08, corresponding to a mean difference of −1.77 (95% CI: −2.05 to −1.50; p < 0.001).

**Figure 3:**
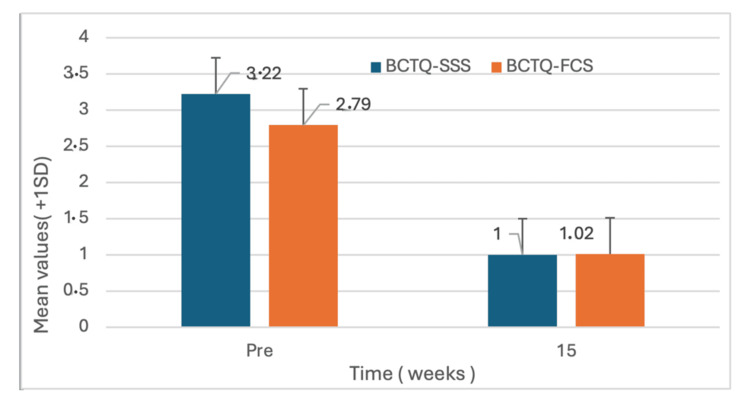
Changes in BCTQ symptom severity and functional status scores from baseline to 15 weeks BCTQ-SSS: Boston Carpal Tunnel Questionnaire-Symptom Severity Scale; BCTQ-FCS: Boston Carpal Tunnel Questionnaire-Functional Status Scale

Sensitivity analysis using non-parametric methods confirmed the same pattern of findings. Statistically significant reductions were observed in BCTQ symptom severity (median difference (IQR): −2.36 (0.82); p < 0.001) and BCTQ functional status (median difference (IQR): −1.87 (1.06); p < 0.001) (Table 3, Figure 3).

Sensitivity analysis for grip and pinch strength

Table 4 presents the non-parametric longitudinal analysis of grip strength and pinch strength across the predefined assessment time points. The values shown in the columns labeled "3 weeks", "9 weeks", and "15 weeks" represent pairwise comparison p-values from Wilcoxon signed-rank tests between each row time point and the corresponding postoperative assessment time point.

**Table 4:** Longitudinal comparison of grip and pinch strength across the predefined assessment time points (non-parametric analysis) Data are presented as median (IQR). The Friedman test was used for overall within-subject comparisons across time points. Values in the columns labeled "3 weeks", "9 weeks", and "15 weeks" represent Bonferroni-adjusted pairwise comparison p-values between the row time point and the corresponding postoperative assessment time point. A p-value of <0.05 was considered statistically significant. IQR: interquartile range

Variable	Time point	Median (IQR)	3 weeks	9 weeks	15 weeks
Grip strength	Preoperative	24.00 (13.5)	0.001	1.000	0.003
	3 weeks	19.00 (13.0)	-	0.001	<0.001
	9 weeks	26.00 (12.0)	-	-	0.002
	15 weeks	32.00 (12.0)	-	-	-
	Friedman test	χ²(3) = 53.71; p < 0.001	Ν/Α	Ν/Α	Ν/Α
Pinch strength	Preoperative	7.40 (2.3)	0.004	1.000	0.001
	3 weeks	6.50 (1.8)	-	0.001	<0.001
	9 weeks	7.40 (2.3)	-	-	0.004
	15 weeks	8.00 (1.9)	-	-	-
	Friedman test	χ²(3) = 55.50; p < 0.001	Ν/Α	Ν/Α	Ν/Α

Statistically significant differences across time were observed for both grip strength (Friedman test: χ²(3) = 53.71; p < 0.001) and pinch strength (Friedman test: χ²(3) = 55.50; p < 0.001).

Pairwise Wilcoxon signed-rank comparisons demonstrated significant differences between all assessment time points, except between the preoperative and nine-week assessments for both variables (adjusted p = 1.000). These results were fully consistent with the parametric analysis and support the robustness of the observed postoperative recovery pattern.

## Discussion

Principal findings

In this prospective observational cohort, patients demonstrated a consistent early decline in grip and key pinch strength at three weeks after standardized mini-open carpal tunnel release, followed by recovery toward baseline by nine weeks and improvement beyond baseline by 15 weeks. This temporal pattern is clinically relevant because it supports the concept that early postoperative weakness after decompression represents an expected phase of recovery rather than treatment failure, provided that symptoms and function subsequently improve.

Importantly, the staged recovery trajectory observed in the present cohort is broadly consistent with both classic and contemporary international literature on postoperative hand strength after carpal tunnel release. Previous studies have shown that grip and pinch strength may decrease during the early postoperative period before gradually recovering over subsequent follow-up, while other longitudinal investigations have similarly described a non-linear pattern of strength restoration after open carpal tunnel release, with initial weakness followed by progressive normalization and later improvement [7-10,21]. Our findings align closely with this established pattern while also providing a clearly predefined early follow-up sequence at three, nine, and 15 weeks.

A clinically meaningful feature of the present study is that preoperative and nine-week strength values did not differ significantly, suggesting that objective strength had largely returned to baseline by that time point, whereas the significant gains observed at 15 weeks indicate continued recovery beyond the early postoperative phase. In parallel, patient-reported symptoms and functional limitations improved substantially by the final follow-up assessment. Taken together, these findings support the value of combining serial objective strength measurements with disease-specific patient-reported outcome measures when characterizing early postoperative recovery after carpal tunnel release, particularly in the context of structured follow-up and standardized postoperative management.

Surgical technique in the context of current literature

All procedures in the present study were performed using a standardized mini-open carpal tunnel release through a limited palmar incision, allowing the direct visualization of the transverse carpal ligament while minimizing unnecessary soft-tissue dissection. This approach is consistent with contemporary limited-incision and mini-open techniques that aim to preserve the advantages of direct visualization while potentially reducing wound-related morbidity compared with more extensive conventional open approaches.

Published comparative studies suggest that mini-open and other limited-incision techniques can achieve symptom relief and patient satisfaction comparable to conventional open carpal tunnel release while in some series reducing scar discomfort, pillar pain, and local wound morbidity [18,19]. In this context, the favorable early functional recovery observed in our cohort is consistent with internationally reported outcomes for contemporary mini-open surgery. However, because the present study was not designed as a comparative surgical trial and did not include an open-release control group, our findings should not be interpreted as evidence of superiority of the mini-open technique itself. Rather, they support that a standardized mini-open approach can provide effective decompression and an early postoperative recovery trajectory that appears comparable to that described in other open and mini-open series.

This distinction is important when interpreting the present findings. The strength of the current study is not a head-to-head comparison between surgical techniques, but rather the detailed characterization of early postoperative recovery within a standardized mini-open cohort, using serial objective strength assessments and disease-specific functional outcomes. Within that framework, our results further support the use of mini-open carpal tunnel release as a clinically valid and contemporary surgical option. The temporal pattern of strength recovery observed in the present mini-open series closely resembles that reported after conventional open release in classic studies of grip and pinch strength recovery [7-10,21], suggesting that our standardized mini-open technique achieves a postoperative course comparable to internationally reported open procedures.

Early functional recovery and hand strength

The time course of strength recovery observed in this study reinforces the view that postoperative restoration of hand function after carpal tunnel release is not linear. Earlier reports have shown that grip and pinch strength often decline in the immediate postoperative period before gradually returning to, and in some cases exceeding, preoperative values over the ensuing weeks and months [7-10,21]. Our finding of a marked decrease in grip and key pinch strength at three weeks, recovery to approximately baseline levels by nine weeks, and improvement beyond baseline by 15 weeks is therefore consistent with the staged recovery pattern described in previous longitudinal studies of strength and dexterity after carpal tunnel release [7-10,21].

This non-linear trajectory is clinically important, as temporary weakness may interfere with functional performance and delay return to daily and occupational activities during the early postoperative period [7-9]. In the present cohort, the absence of a statistically significant difference between preoperative and nine-week strength values suggests that objective strength had substantially normalized by that stage, whereas the significant improvement at 15 weeks supports the interpretation that recovery continued beyond simple return to baseline. This pattern may reflect a combination of reduced neural compression, progressive restoration of median nerve function, improved tolerance to hand use, and gradual recovery of force production after the early postoperative phase.

The parallel improvement observed in both objective strength measures and disease-specific questionnaire scores further supports the interpretation that early weakness should be understood as part of the normal postoperative course after adequate decompression rather than as evidence of persistent dysfunction in most uncomplicated cases. For this reason, the combined use of serial grip and key pinch measurements with validated patient-reported outcome tools may provide a more clinically informative assessment of recovery than reliance on symptoms or strength alone [11-17].

Influence of postoperative management and rehabilitation

Interpretation of early recovery after carpal tunnel release remains challenging because the published literature is heterogeneous with respect to surgical technique, follow-up timing, postoperative splinting practices, rehabilitation content, and outcome selection [7-10,18-25]. This heterogeneity is a major reason why direct comparison across studies remains difficult, particularly when evaluating the relative contributions of surgical decompression, natural biological recovery, and structured postoperative rehabilitation.

International evidence has increasingly questioned the routine use of prolonged postoperative immobilization after carpal tunnel release. Randomized studies and later reviews have reported little or no consistent benefit of routine splinting for pain reduction or functional recovery, while some studies suggest that unnecessary immobilization may delay return to usual hand use [20,22-24]. In contrast, early mobilization is generally considered compatible with uncomplicated recovery after adequate decompression. Our postoperative management, which avoided routine immobilization beyond the immediate postoperative phase and encouraged early hand use, is therefore aligned with contemporary literature and current clinical reasoning.

With respect to rehabilitation, some studies have suggested that targeted hand rehabilitation may support postoperative recovery of grip strength and functional performance, but the magnitude, consistency, and independent contribution of these effects remain uncertain across the literature [25]. In the present study, all participants followed the same structured home-based strengthening program beginning at the third postoperative week, which provided a standardized rehabilitation context during follow-up. However, because no comparison group receiving alternative management or no structured exercise was included, the present findings should not be interpreted as evidence that the exercise protocol alone was responsible for the observed improvement. Rather, our results indicate that recovery occurred within the context of standardized early mobilization and progressive home-based loading, following a trajectory that remains consistent with internationally reported postoperative recovery patterns.

This interpretation is particularly important for clinical applicability. The current study supports the feasibility of a simple, structured home-based postoperative strengthening strategy after the initial wound-healing phase, but it does not establish superiority over natural recovery or other rehabilitation models. Future comparative studies will be necessary to determine the independent therapeutic contribution of specific exercise protocols after carpal tunnel release.

Clinical implications

From a practical standpoint, the present findings may assist clinicians in setting realistic expectations after carpal tunnel release. Patients can be informed that a temporary decline in grip and key pinch strength during the early postoperative weeks is compatible with the expected recovery course, particularly when symptom burden and functional status improve in parallel. This may help reduce unnecessary concern during early follow-up and may improve adherence to staged return-to-activity recommendations.

In addition, the combination of serial grip and key pinch measurements with a validated disease-specific questionnaire such as the BCTQ offers a clinically useful framework for postoperative monitoring. This combined approach may help identify patients who are following the expected recovery trajectory versus those with delayed recovery, persistent weakness, or disproportionate functional limitation who may benefit from closer reassessment or more individualized rehabilitation planning.

Limitations and future directions

This study has limitations inherent to its prospective observational design, which precludes causal inference regarding the isolated effects of the postoperative exercise protocol on strength recovery. The sample size was relatively limited, no formal a priori power calculation was performed, and follow-up focused on the early to intermediate postoperative period, all of which may reduce the generalizability of the findings.

Functional outcomes using the BCTQ were assessed only at baseline and at 15 weeks, without intermediate patient-reported assessments, potentially overlooking finer temporal changes in symptoms and function. Furthermore, no external control or comparison group and no systematic comparison with the contralateral hand were included, which limits the ability to distinguish the contribution of structured exercise from natural postoperative recovery and to contextualize strength changes relative to each patient's overall hand function. Formal adherence to the home-based exercise program was not quantified, and electrodiagnostic severity grading was not incorporated into the present analysis.

Despite these limitations, the pattern of strength recovery and symptom improvement observed in our mini-open series appears consistent with that reported in previous open and mini-open studies [7-10,18-21,23,25]. Future research with larger samples, longer standardized follow-up, and randomized or comparative rehabilitation designs across different surgical techniques will be important to clarify the independent contribution of structured postoperative exercise and to further define optimal strategies for early functional recovery after carpal tunnel release.

## Conclusions

In this prospective observational cohort, grip and key pinch strength showed a consistent pattern of early postoperative decline at three weeks after carpal tunnel release, recovery toward baseline by nine weeks, and improvement beyond baseline by 15 weeks. Patient-reported symptom severity and functional status also improved substantially by the final follow-up. These findings support the value of serial objective strength testing combined with patient-reported outcome measures in characterizing early postoperative recovery after carpal tunnel release. However, the findings should be interpreted cautiously given the observational design, limited sample size, and absence of an external comparator group. Further studies with larger samples and comparative rehabilitation designs are needed to better define the independent contribution of structured postoperative exercise to recovery.
